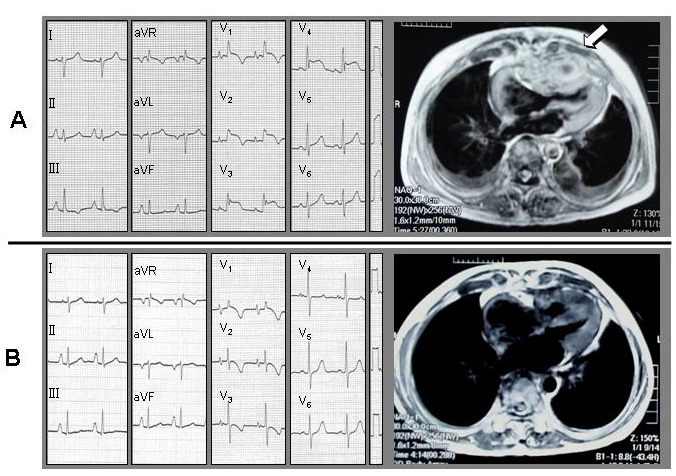# Brugada-like Precordial ST Elevation on ECG by Anterior Mediastinal Infective Mass Lesion

**Published:** 2003-07-01

**Authors:** Yuji Nakazato, Takayasu Ohmura, Issei Shimada, Hiroyuki Daida

**Affiliations:** Division of Cardiology, Department of Internal Medicine, Juntendo University School of Medicine, Tokyo, Japan

Several causes are known to induce the right precordial ST elevation mimicking Brugada syndrome. Right ventricular outflow area is assumed to be responsible for such ECG changes. We experienced a case of anterior mediastinal infective mass lesion with a Brugada-like ECG.

A 52-year-old female, who has pulmonary stenosis and recurrent episodes of right ventricular heart failure, complained of high fever, abdominal discomfort, and edema. On physical examination, jugular vein dilation, hepatomegaly, and facial and leg edema were noted. Leucocytosis was also noted on blood examination. An ECG showed right ventricular hypertrophy, incomplete right bundle branch block pattern and marked ST elevation on precordial leads mimicking Brugada syndrome. Magnetic resonance imaging revealed an abnormal mass shadow located on the anterior mediastinum and compressing the right ventricle (Figure 1A). Trans-thoracic echocardiography also showed the high echogenic mass lesion at the anterior side of right ventricle and the vicinity of pulmonary valve. After treatment with antibiotics, the mass lesion gradually shrunk. Concomitantly, the ST elevation disappeared with improvement of inflammatory markers (Figure 1B). The symptoms suggesting right ventricular failure were also ameliorated. The mechanism of Brugada-like ST elevation in this patient was considered to be compression, by the abnormal infective mass, of the right ventricular outflow tract with/without focal pericardial inflammation.

## Figures and Tables

**Figure 1 (A and B):**